# Concurrent müllerianosis of the urinary bladder and the umbilicus presenting with umbilical bleeding: a rare case report and review of the literature

**DOI:** 10.1186/s12905-020-00929-3

**Published:** 2020-04-03

**Authors:** Syu Jhang, Marcelo Chen, Li-Chen Chen

**Affiliations:** 1 Department of Urology, Mackay Memorial Hospital, No. 92, Sec. 2, Zhongshan N. Rd, Taipei, 10449 Taiwan; 2 School of Medicine, Mackay Medical College, Sanzhi, Taiwan; 3 Mackay Junior College of Medicine, Nursing and Management, Taipei, Taiwan

**Keywords:** Müllerianosis, Umbilicus, Urinary bladder

## Abstract

**Background:**

Müllerianosis is a very rare neoplasm composed of two or three Müllerian derived tissues (endosalpinx, endometrium and endocervix). We report the first case of concurrent müllerianosis of the urinary bladder and the umbilicus presenting with umbilical bleeding.

**Case presentation:**

A 43-year-old Asian premesopausal female, gravida 1, para 1, presented with intermittent umbilical bleeding. An umbilical nodule and a bladder tumor on the posterior wall of the urinary bladder were identified. She underwent transurethral resection of the bladder tumor and excision of the umbilical nodule successively. Diagnosis of müllerianosis was confirmed by the histological and immunological features. No tumor recurrence was noted at 6 months of follow-up.

**Conclusions:**

Müllerianosis is extremely rare and mainly reported in the urinary bladder, and generally affects women of reproductive age. Despite the common presentations of müllerianosis of the urinary bladder including irritative voiding symptoms, abdominal/pelvic pain and gross hematuria, our rare case had no symptom except umbilical bleeding. The possibility of concurrent bladder müllerianosis should be considered when müllerianosis is found at other location. We suggest a surgical intervention to establish the correct pathological diagnosis because it is essential to exclude malignant neoplasms of the urinary bladder. The majority of patients have a favorable prognosis.

## Background

Müllerianosis is a very rare neoplasm composed of at least two of müllerian-derived tissues, namely endosalpinx, endometrium and endocervix, [1] and has mainly been reported in the urinary bladder. Only approximately 20 cases of müllerianosis of the urinary bladder have been documented in the English literature [2]. Herein, we report the first case of concurrent müllerianosis of the urinary bladder and the umbilicus presenting with umbilical bleeding.

## Case presentation

**Fig. 1:**
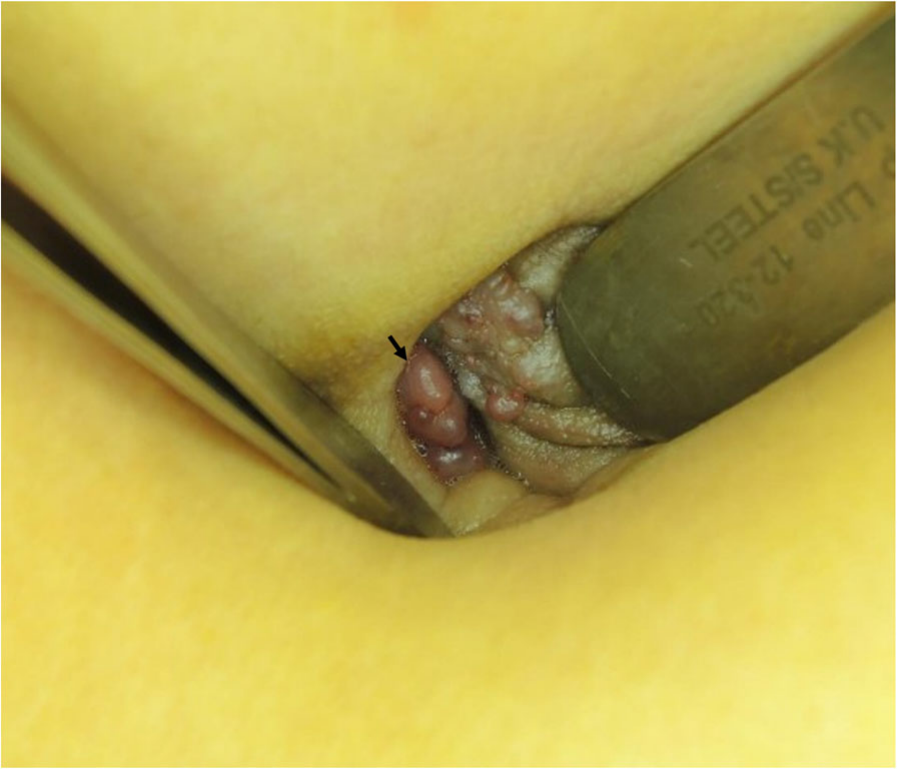
A firm, irregular and unmovable umbilical mass with intermittent bleeding (arrow)

**Fig. 2:**
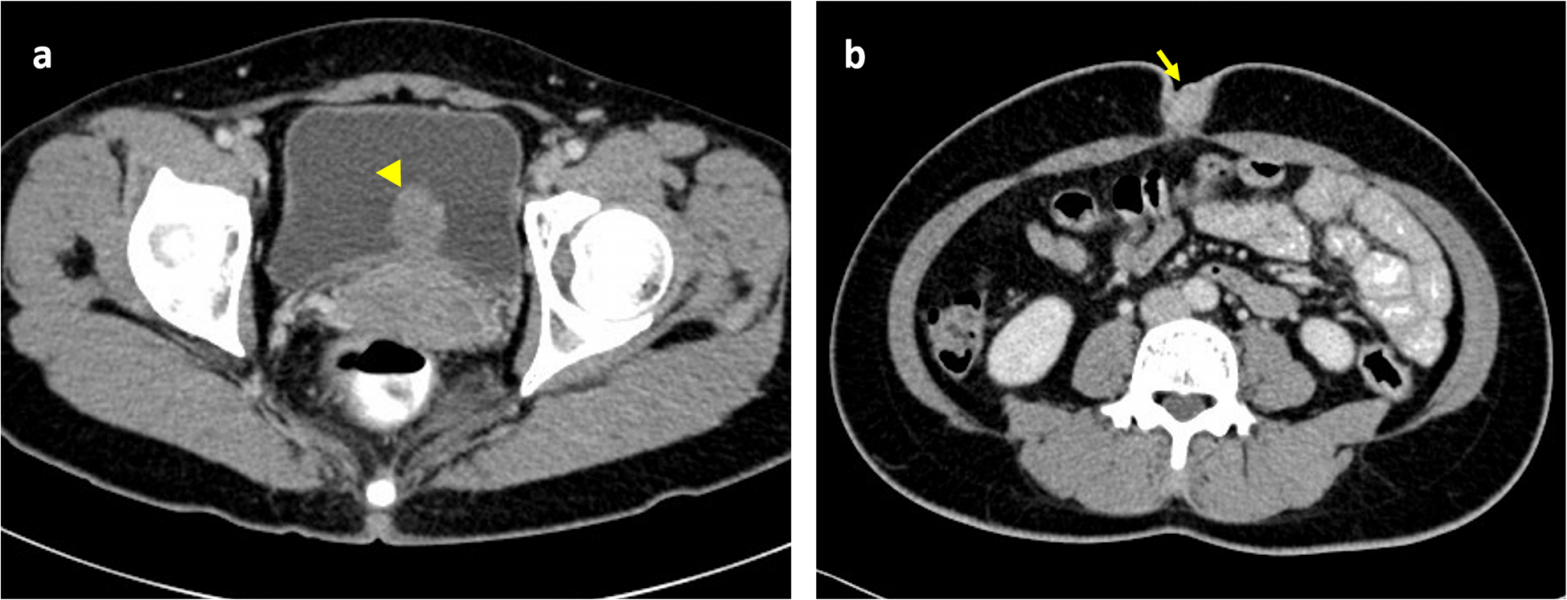
Computed tomography revealing **a**. A protruding mass on the posterior wall of the urinary bladder (arrowhead). **b**. A mass in the umbilicus (arrow)

**Fig. 3:**
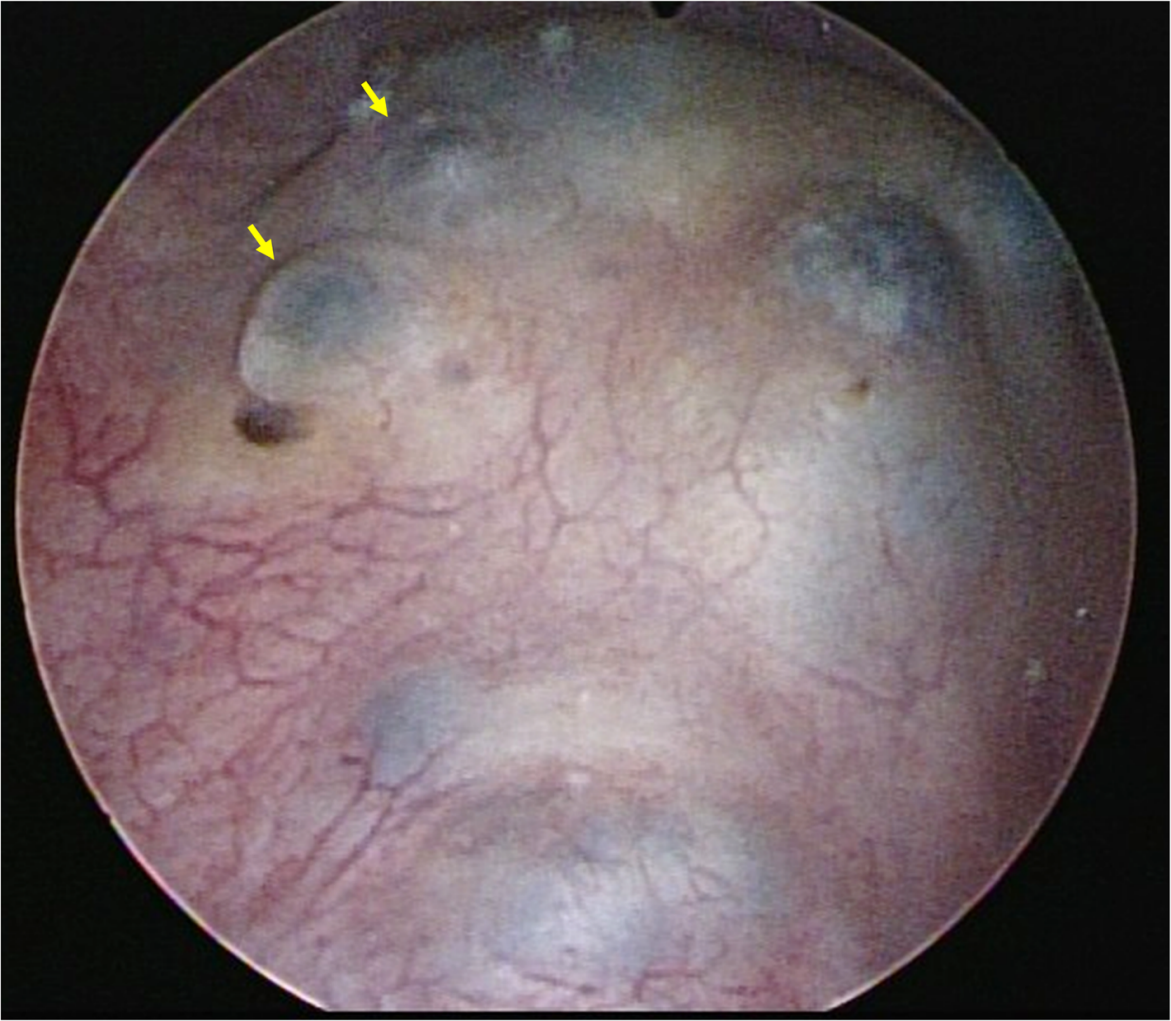
Cystoscopy revealing a protruding mass with hemorrhage underneath the intact epithelium on the posterior wall of the urinary bladder (arrow)

**Fig. 4:**
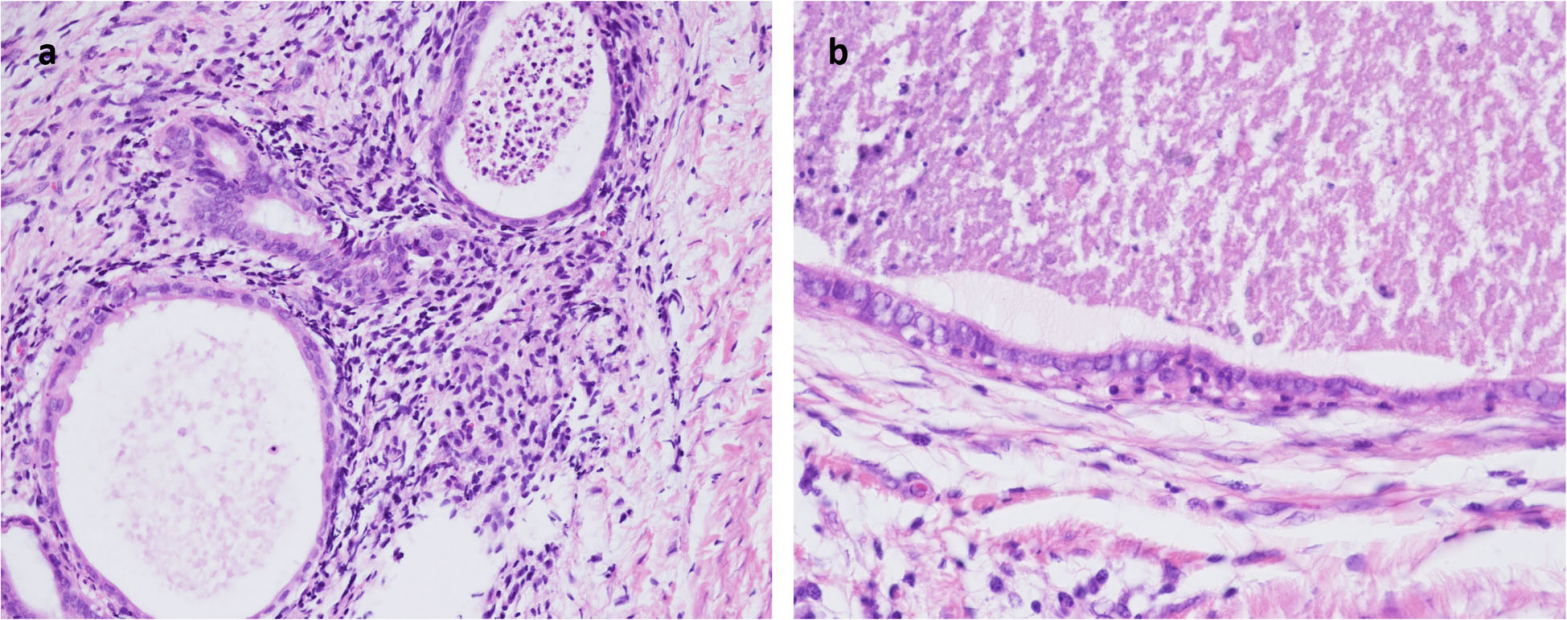
Microscopic histological examination revealing **a**. Glands with dilated lumen and surrounding short spindle cells consistent with endometriosis. **b**. Glands with no spindle cell but cilia consistent with endosalpinx

**Fig. 5:**
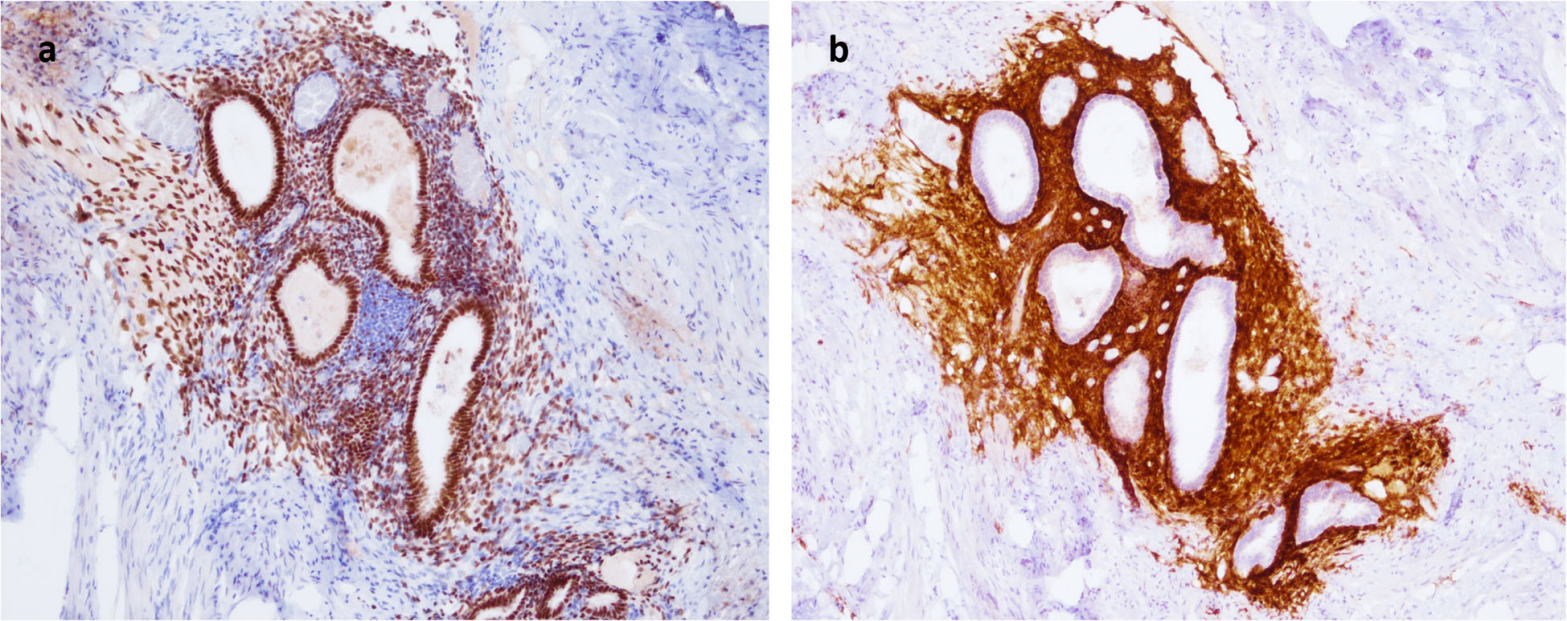
Positive stains for **a**. Estrogen receptor. and **b**. CD10

A 43-year-old Asian premesopausal female without known underlying disease or history of surgery, gravida 1, para 1, complained of intermittent umbilical bleeding for several weeks. She had a normal body mass index (24.1 kg/m^2^). She had no other symptoms including abdominal/pelvic pain, gross hematuria, urinary frequency, micturition pain, dysmenorrhea or dyspareunia. On pelvic examination, she had no lifting tenderness. She visited our Obstetrics and Gynecology clinic, where a bladder lesion was incidentally identified on transabdominal ultrasound. A firm, irregular and unmovable nodule was also noted in the umbilicus with a diameter of 2 cm (Fig. 1). She had no lifting pain Urinalysis showed 6 red blood cells per high-power field. Abdominal computed tomography revealed a protruding mass 2.4 cm in diameter located on the posterior wall of the urinary bladder (Fig. 2a) and another separate mass in the umbilicus (Fig. 2b) without communication between these two lesions. She first underwent transurethral resection of the bladder tumor (TURBT) (Fig. 3), and a microscopic histological examination revealed multiple foci of glands with dilated lumen and surrounding short spindle cells. The glands were composed of bland-looking cells with a tall columnar shape (Fig. 4a). Immunohistochemically, estrogen receptor (ER) and CD10 were expressed in the surrounding short spindle cells (Fig. 5a and b). Cilia and ER expressions were also noted in dilated glands without spindle cell cuffing (Fig. 4b). These histological findings were consistent with endometrium and endosalpinx, and a diagnosis of müllerianosis was made. Occult malignancy was not seen. She had no discomfort after the surgery except for intermittent umbilical bleeding. Thus, excision of the umbilical tumor was performed. The pathological finding was also compatible with müllerianosis. Physical examination and cystoscopy at 6 months of follow-up revealed no tumor recurrence.

## Discussion and conclusions

Young and Clement [3] reported the first case of müllerianosis in the urinary bladder in 1996 and defined this disease as “a lesion seen at any site containing admixtures of endosalpingiosis, endometriosis, and endocervicosis.” Twenty-one cases occurring in the urinary bladder have been documented in 19 articles (Table 1) in the English literature, accounting for the majority of cases of müllerianosis [18]. Some sporadic cases have been reported in the ureter, spinal cord, inguinal lymph nodes and mesosalpinx [2]. To the best of our knowledge, this is the first report to describe müllerianosis of the umbilicus. Due to its rarity, the prevalence of müllerianosis is unknown.

**Table 1:** Review of reported cases of müllerianosis of the urinary bladder

Case Number	Age	Symptoms	Operation history	Tumor location	Treatment	Follow up	Reference
1	37	N/A	C/S (one of these three)	P	TURBT	N/A	[3]
2	44	Lower abdominal pain		P	TURBT	N/A	[3]
3	46	Irregular menstruation		P	TURBT	N/A	[3]
4	27	Dysmenorrhea	None	P	N/A	N/A	[4]
		Irritative voiding symptom^a^ (dysuria)					
5	38	Pelvic pain	Hysterectomy	N/A	PC	N/A	[5]
		Irritative voiding symptom (dysuria)					
6	48	Lower abdominal pain	Hysterectomy	N/A	Biopsy	Persistent but significantly improved symptoms at 3 months	[6]
		Dyspareunia					
		Irritative voiding symptom (frequency/urgency)					
		Hematuria					
7	37	Vaginal discharge	C/S	P	TURBT	N/A	[7]
		Iliac fossa pain	SO				
8	53^b^	Irritative voiding symptom (dysuria)	N/A	P	TURBT	N/A	[8]
		Hematuria					
9	41	Irritative voiding symptom (dysuria)	None	P	TURBT	N/A	[9]
		Pelvic pain					
		Hematuria^a^					
10	70^b^	Vaginal bleeding	Hysterectomy	Trigone	N/A	N/A	[10]
			SO				
11	45	Pelvic pain	C/S	P	Biopsy and PC	Complete symptoms relief	[11]
		Dysmenorrhea					
		Irritative voiding symptom					
		Hematuria^a^					
12	32	Irritative voiding symptom^a^ (dysuria)	N/A	Right lateral wall	TURBT and GnRH analogue	Persistent but smaller tumor at 2 years	[12]
13	28	Hematuria	None	Dome and left lateral wall	TURBT	No tumor recurrence at 6 months	[1]
14	50	Renal colic	Hysterectomy	Left UVJ	TURBT and GnRH analogue	Partial symptoms relief and smaller tumor at 3 months	[13]
		Dysmenorrhea					
		Pelvic pain.					
15	61^b^	Irritative voiding symptom (dysuria/frequency)	C/S	P	TURBT	N/A	[14]
16	50	Hematuria	None	P	TURBT	N/A	[15]
		Irritative voiding symptom (dysuria)					
17	30	Iliac fossa pain	None	P	TURBT and PC	No tumor recurrence at 1 month	[16]
18	39	Irritative voiding symptom^a^ (dysuria)	None	P	TURBT	N/A	[17]
19	64^b^	Lower abdominal pain	C/S	Left UVJ	TURBT and PC	Complete symptoms relief	[18]
		Repeat UTI	Hysterectomy				
		Hematuria	Appendectomy				
20	59^b^	Repeat UTI	None	Dome	TURBT twice	Tumor recurrence and need PC	[19]
		Suprapubic pain					
		Hematuria					
		Irritative voding symptom (dysuria/incontinence)					
21	40	Irritative voiding symptom^a^ (urgency/incontinence)	N/A	P	Biopsy	N/A	[2]
22	43	Umbilical bleeding	None	P	TURBT	No tumor recurrence at 6 months	Present case

*N/A* not available; *C/S* Cesarean section; *P* posterior wall; *TURBT* transurethral resection of the bladder tumor; ^a^ symptoms occur cyclically or during the menstrual period; *PC* partial cystectomy; *SO* salopingo-oophorectomy; ^b^ = postmenopausal patient; *GnRH* Gonadotropin-releasing hormone; *UVJ* ureterovesical junction; *UTI* urinary tract infection

Several hypotheses of the pathogenesis of müllerianosis of the urinary bladder have been proposed; however, the mechanism is not clearly understood. Implantation, one of the most discussed theories, suggests that müllerian tissue implants into the urinary bladder wall during pelvic surgery [3]. However, this cannot explain why müllerianosis is found in surgery-naïve patients or in organs other than the urinary bladder. Ten of the 19 cases (52.6%) with detailed surgical records, the present case included, had not experienced any pelvic surgery such as hysterectomy or cesarean delivery. Another theory, metaplasia, advanced by Donne et al., [4] is based on the presence of two or more müllerian-derived tissues, which implies the potential of müllerian epithelium to differentiate rather than the implantation of a single type of the tissue. Moreover, of the 20 cases with marked tumor locations, a total of 16 tumors (80%) were situated on the posterior wall or the dome of the urinary bladder, a place adjacent to the peritoneum and more sensitive to female hormones. Koren et al. [9] in 2006 reported a case supporting the metaplasia theory with the metaplastic epithelium in continuity with the urothelium. The present case is also supportive of metaplasia because of concurrent occurrence of the bladder and the umbilical masses.

Müllerianosis of the urinary bladder mainly affects women of the reproductive age, with a mean age at diagnosis of 44.6 years (range 27 to 70 years). Of the total 22 patients with müllerianosis of the urinary bladder, five were postmenopausal females. The most common symptoms have been reported to be irritative voiding symptoms (54.5%) including frequency, urgency, urge incontinence and dysuria, abdominal/pelvic pain (45.5%) and gross hematuria (36.4%). These symptoms occurred cyclically in six cases (27.3%), mostly during the menstrual period. One patient with a tumor on the ureterovesical junction presented with renal colic [13]. Our case had none of the symptoms associated with bladder lesions, and only had umbilical bleeding.

Grossly, müllerianosis of the urinary bladder has been described as “sessile polypoid”, [17] “submucosal”, [6] or “smooth with normal appearing overlying mucosa” [14] on cystoscopic examinations. The gross appearance of the bladder tumor in our case revealed the similar findings as a mass with hemorrhage underneath the intact epithelium. Histologically, it revealed multiple foci of glands with variable size lined by tubal, endocervical, or endometrial epithelium situated in the lamina propria and muscularis propria [2, 15]. These glandular cells were immunohistochemically positive for ER and progesterone receptor (PR) [9]. In addition, the stroma surrounding the endometrial glands diffusely expressed CD10, and the epithelia showed positive staining for Ca-125 [15].

Both medical and surgical treatments can be used for müllerianosis of the urinary bladder. Regardless of the lack of consensus on the choice of therapy, most physicians and patients chose the surgical intervention in the reported cases, and most of the patients had a favorable prognosis. Sixteen patients underwent TURBT, only five of whom required subsequent medical treatment, [12, 13] or partial cystectomy [16, 18, 19] due to persistent symptoms or tumor recurrence. None of the four patients who underwent partial cystectomy had recurrence; by contrast, in two cases using gonadotropin-releasing hormone (GnRH) analogues to inhibit pituitary and gonadal function to reduce the tumor volume as medical treatment, cystoscopy showed smaller but persistent tumors at 3 months and 2 years of follow up. There was no documented distant metastasis or mortality in the cases with short-term follow-up (1–24 months).

Müllerianosis of the urinary bladder mimics several bladder neoplasms such as cystitis glandularis and nephrogenic adenoma [9]. It is most important to differentiate between müllerianosis and invasive adenocarcinoma, both of which exist in the lamina propria and muscularis propria. A case of bladder endometrioid adenocarcinoma complicating müllerianosis has been reported [8].

In conclusion, müllerianosis is a rare neoplasm which mainly affects women of reproductive age. The common presentations of müllerianosis of the urinary bladder include irritative voiding symptoms, abdominal/pelvic pain and gross hematuria. In our case, multifocal müllerianosis were separately located on the urinary bladder and the umbilicus, which implies that the possibility of concurrent bladder müllerianosis should be considered when müllerianosis is found at other location. Despite a favorable prognosis in the majority of patients, in view of the importance of establishing the correct pathological diagnosis, we suggest treatment with a surgical intervention consisting of TURBT and partial nephrectomy if recurrence occurs with persistent symptoms.

## Data Availability

The data used and analyzed during the current study are available from the corresponding author on reasonable request.

## Abbreviations

ER: Estrogen receptor
PR: Progesterone receptor
TURBT: Transurethral resection of the bladder tumor
GnRH: Gonadotropin-releasing hormone
